# Attitude and Awareness Toward General and Professional Ethics Among Pharmacists and Pharmacy Students: A Cross-Sectional Study from Jordan

**DOI:** 10.3390/healthcare13131556

**Published:** 2025-06-30

**Authors:** Mariam Alameri, Ghaith Al-Taani, Mervat Alsous, Sireen Shilbayeh, Nadia Al Mazrouei

**Affiliations:** 1 Department of Clinical Pharmacy and Pharmacy Practice, Faculty of Pharmacy, Yarmouk University, Irbid P.O. Box 566, Jordan; g.altaani@yu.edu.jo (G.A.-T.); mervat.alsous@yu.edu.jo (M.A.); 2 Department of Pharmacy Practice, College of Pharmacy, Princess Nourah bint Abdulrahman University, Riyadh P.O. Box 84428, Saudi Arabia; ssabdulrahim@pnu.edu.sa; 3 Pharmacy Practice and Pharmcotherapeutics Department, College of Pharmacy, University of Sharjah, Sharjah P.O. Box 27272, United Arab Emirates; nalmazrouei@sharjah.ac.ae

**Keywords:** attitude, awareness, general and professional ethics, pharmacists, pharmacy students, Jordan

## Abstract

Background: Due to their participation in patient care, pharmacists frequently face various ethical decisions that must be made. Every day, pharmacists encounter ethical challenges in any pharmaceutical workplace. This study aims to assess the attitude and awareness among pharmacy students and pharmacists regarding general and professional ethics. Methods: This study was cross-sectional, using an interview-based questionnaire. Pharmacy students from Yarmouk University volunteered to approach other pharmacy students and pharmacists to complete the questionnaire using a convenient sampling method. Results: A total of 1737 pharmacy students and pharmacists participated in this study. Among the 691 participants who are currently practicing pharmacists, 549 (79.4%) reported working in a community pharmacy. About half of the pharmacy students (53.6%) had studied a course in ethics, while 110 (15.9%) of the pharmacists had not. While students and pharmacists have shown significantly different attitudes, less than 20% of the participants demonstrated a high level of awareness regarding the pharmacy code of ethics. On the other hand, more than 60% of pharmacy students and practicing pharmacists concurred that they might permit others to carry out the duties of a pharmacist. Surprisingly, the data revealed that student pharmacists performed better than practicing pharmacists, highlighting that students appreciate the principles of professionalism and that exposure to a work environment can help modulate negative behavior and beliefs. Conclusions: These findings underscore the need for ongoing ethical education and training for both pharmacy students and practicing pharmacists to ensure that everyone in the field upholds a high standard of moral behavior in patient care.

## 1. Introduction

A key element of ethical decision-making is ethical sensitivity, which refers to a person’s tendency to direct them in moral judgments [1]. Excellent ethical judgment accompanies high ethical awareness. On the other hand, a lack of ethical awareness can lead to unethical behavior, which can harm both individuals and the workplace [2]. Due to their participation in patient care, pharmacists frequently face various ethical decisions that must be made. Every day, pharmacists encounter ethical challenges in any pharmaceutical workplace. As the pharmacist’s duties increase, they will soon encounter ethical issues that other healthcare professionals working in clinical settings also face, which will lead to increased levels of moral anguish [3,4]. These can range from straightforward problems, such as choosing the best product for a patient, to complex ethical dilemmas, like balancing moral principles and legal requirements when a patient requests an illegal medication without a prescription but is in urgent need [5]. It has been suggested that pharmacy students undergo thorough ethical instruction during their studies, both before and after long-term assignments, particularly in hospital and pharmacy settings, to address these issues [6]. Over the past 20 years, the focus of pharmacy practice has shifted from dispensing prescriptions to fostering closer connections with patients and placing greater emphasis on pharmacists’ accountability for achieving successful patient outcomes [7]. As a result, like other healthcare professionals, pharmacists are accountable for providing the highest quality services in healthcare [8]. Pharmacists should work closely with patients as healthcare providers and in collaboration with insurance companies [9]. As essential members of the healthcare team, pharmacists have a professional and ethical responsibility to protect patient health, ensure that drugs are used sensibly, and advance the general welfare. These duties are governed by a code of ethics enforced by national regulatory organizations like the Jordan Pharmacists Association (JPA), which is in line with global norms like those set forth by the World Health Organization (WHO) and the International Pharmaceutical Federation (FIP) [10]. This code, which emphasizes values like beneficence, non-maleficence, autonomy, and justice, provides a framework for moral decision-making and professional behavior. In Jordan, the JPA code of ethics is usually taught in pharmaceutical law or professional practice courses for undergraduate students. Teaching pharmacy ethics has been ongoing for a long time, and it is not yet up to par [11]. However, different institutions may differ in how thoroughly and consistently they teach and explain the instructions, and this may not fully prepare students for problems they may face in the real world [12]. Additionally, practicing pharmacists have few opportunities for continuous professional development (CPD) in pharmacy ethics, and there isn’t a national system in place that requires constant ethical training after graduation [13]. This calls into question how equipped pharmacists are to handle changing ethical dilemmas in their line of work.

It is crucial to comprehend students’ and pharmacists’ awareness of ethical standards from a professional perspective. It draws attention to current deficiencies in education and training and can help guide the creation of curricula and policy choices that will enhance ethical competency at every level of a pharmacist’s career, since few studies have been done on pharmacy ethics [14], and the literature contains very little research examining ethical sensitivity among pharmacy students [15,16]. Thus, the purpose of this study is to shed light on the attitudes and awareness among pharmacy students and pharmacists regarding general and professional ethics. By doing this, it aims to pinpoint the ethical training’s strong and weak points, supporting initiatives to raise the bar for pharmacy practice ethics.

## 2. Methodology

This was a prospective cross-sectional study that used an interview-based questionnaire. Pharmacy students from Yarmouk University volunteered to approach other pharmacy students and pharmacists to complete the questionnaire using a convenient sampling method.

University pharmacy students, fresh graduate pharmacists with work experience of less than one year, and graduate pharmacists with work experience of more than one year were approached between November 2023 and May 2024.

After reviewing the literature, the questionnaire was adapted mainly from three studies [17,18,19]. Afterward, it was reviewed by experts to ensure the clarity of the questions. An Arabic-language questionnaire was built using the back-translation method. A pilot study involving 20 subjects was undertaken to examine the final draft of the survey.

An introductory paragraph was created on the first page to ensure the confidentiality and anonymity of the data collected. The participants consented using the statement, “I consent to participate in the study”, after the introductory paragraph, which the participant must agree to before proceeding to the first question.

The institutional review board approval from Yarmouk University is obtained with a reference number (IRB/2023/530).

### 2.1. Sample Size

For this observational study, the following equation for an unlimited population was used to determine the sample size:

$$n=(z^{2}\times \hat{p}(1-\hat{p}))\;/\;\varepsilon^{2}$$

where n is the sample size, z is the statistic corresponding to the level of confidence, for a 95% confidence interval, z is 1.96, p is the expected prevalence (results from similar studies), and d is precision, d = 0.05. This equation results in the largest sample size when p = 0.50 and 1 − p is 0.50. By using the above values, the sample size is 380 participants. However, a larger sample size was targeted to ensure greater representativeness.

### 2.2. Statistical Analysis

Data was analyzed using IBM SPSS statistics software version 23. Yarmouk University has an institutional license for the IBM SPSS statistical software utilized in this study. Descriptive analysis, including frequency and percentage for categorical variables. Mean ± standard deviation for continuous data. The chi-square test was used to measure the differences between categorical variables. Cronbach’s alpha and the interclass correlation coefficient were used to measure the reliability of the measurements used in the study.

## 3. Results

### Sample Characteristics

Data were analysed from 1737 pharmacy students and pharmacists in Jordan. The mean age was 22.26 ± 2.84 and 29.06 ± 7.43 for students and pharmacists, respectively. Most participants were female and from public universities, including both pharmacy students and pharmacists.

About half of the pharmacy students had studied a course about ethics, while 110 (15.9%) of the pharmacists had not studied a course about ethics. Regarding the period of pharmacy training, 352 (33.7%) of students had no training, while 270 (25.9%) had training for more than 4 months. 

Only 83 (12%) of pharmacists work in the pharmaceutical industry, while 549 (79.4%) work in community pharmacy. One-third of the pharmacists had work experience in the pharmacy field. Most participants reported that the ethics course they had taken addressed all elements of ethics. The results of demographic data are provided in Table 1.

The majority of students and pharmacists held the following beliefs: that morality and ethics were crucial to their profession, that being truthful was the best policy when interacting with patients and other medical professionals, and that corruption was illegal, even when it was most beneficial to society. More than half of the participants disagreed that lying is acceptable to survive and that outcomes take preference over politeness. When pharmacists’ and students’ perspectives were compared, the pharmacists’ views were more discordant, believing that results come before manners, that corruption is acceptable, and that lying is necessary to survive (*p*-value < 0.05). Table 2 displays the findings.

Less than 20% of the participants demonstrated a high level of awareness regarding the pharmacy code of ethics. They also committed to telling the truth to the patient, even if the treating physician withheld information about adverse drug information, and that it is not a good idea to share patient information with friends and family. Furthermore, they acknowledged that it is possible to conduct promotional activities in pharmacies and provide recommendations for non-pharmaceutical health products, in addition to prescription drugs, within their pharmacies.

On the other hand, more than 60% of pharmacy students and practicing pharmacists concurred that they might permit others to carry out the duties of a pharmacist, that profit is the pharmacy’s primary goal and should take precedence over keeping ethical standards in the workplace, that they can dispense prescription drugs without a prescription and that they would dispense a medication that has an error without contacting the doctor.

Unfortunately, a significant number of participants stated that they could disparage the skills of other healthcare professionals and that they may dispense an OTC medication that the customer requested, even though they have concerns about the drug being abused. Moreover, they can provide natural products with unproven safety and efficacy if requested by the patient.

Univariate analysis revealed that participants working in community pharmacies had lower awareness of the pharmacy code of ethics compared to those working in other pharmaceutical fields (*p*-value < 0.001).

Compared to students, a larger proportion of pharmacists stated that a pharmacist doesn’t need to be present at the pharmacy all the time, that they may delegate the task of being a pharmacist to others, that they would dispense a prescription that has an error without contacting the doctor, that they would substitute medications with alternatives drugs with lower cost, that they could provide non-medical products as well as natural products with undocumented safety and efficacy if requested by the patient, and that they may dispense an OTC medication that the customer requested even though they have concerns about him abusing it.

Moreover, a higher percentage of pharmacists disagreed that it is possible to have promotional activities in the pharmacy and that it is crucial to notify patients of all relevant information, including side effects of medications, even if the treating physician chooses to withhold it from them and not share patients’ information with patients’ relatives and friends, and that it is possible to have promotional activities in the pharmacy and that it is crucial to notify patients of all relevant information, including side effects of medications, even if the treating physician chooses to withhold it from them and not share patients’ information with patients’ relatives and friends.

On the other hand, a larger proportion of students reported having a good awareness of the pharmacy code of ethics compared to pharmacists and disagreed that they can dispense a prescription drug without a prescription (*p*-value < 0.05). Results are shown in Table 3.

## 4. Discussion

The purpose of this study was to evaluate and compare the attitudes and awareness among pharmacy students and pharmacists regarding general and professional ethics in Jordan. The results showed that student pharmacists adhered to ethical principles more strongly, underscoring the ethical issues that are currently prevalent in community pharmacy practice.

Ethical principles are essential for the professional practice of pharmacy. These ethical principles include, among others, beneficence, truth-telling, privacy, and respect for the person [20]. However, the utilization of these ethical principles in pharmacy practice is not automatic. Despite the intention to concur with ethical principles, several ethical dilemmas can prevail where two or more conflicting ethical principles and other beliefs are competing, which might have a negative impact on one party [21]. The present study surveyed community pharmacists and student pharmacists in Jordan regarding ethical principles and dilemmas, and identified issues with certain items, such as disclosing information to patients without the treating physician’s recommendation, dispensing medications in unethical manners, and prioritizing results over manner. Unfortunately, when the results were compared between student pharmacists and community pharmacists, the data revealed that student pharmacists performed better than practicing pharmacists, highlighting that students appreciate the principles of professionalism and that being exposed to a work environment can modulate negative behavior and beliefs. Such results highlighted the ethical issues prevailing in the community pharmacy setting, which would guide the development of further strategies to improve the status quo. 

Recent developments in pharmacy education in Jordan have resulted in a greater focus on ethics due to organized coursework, the incorporation of ethical case studies from real-world situations, and the encouragement of professional values in academic contexts. As a result, students may have a more up-to-date and theoretically based ethical awareness than professional pharmacists, who might not have had the same amount of formal training throughout their school years [14]. Moreover, students’ ethical views and awareness might be shaped by the rising exposure to ethical debates, public health initiatives, and professional role models they receive through social and digital media platforms. In academic settings, peer relationships serve to further solidify idealistic standards of professional behavior [22]. Although the Jordanian Pharmacists Association has ethical guidelines, most pharmacists in Jordan do not use them [11]. Additionally, ethics-related courses are not often required in continuing professional development (CPD) programs, and there are still insufficient safeguards against unethical behavior in public places. In professional settings where commercial pressures or a lack of institutional support may compromise ethical standards, this regulatory gap may eventually lead to ethical drift [23].

An important, critical, and essential ethical principle is truth-telling, which is highly relevant to decision-making in healthcare. In the present study, a high extent of pharmacy students and pharmacists were accustomed to telling the truth to the patient and healthcare professionals. This behavior can build trust between the pharmacist and patient and respect the patient and his/her autonomy, which is reflected in following the pharmacist’s advice regarding the treatment plan [24,25]. Sometimes, hard healthcare professionals might not disclose complete information to patients, for instance, regarding bad news or complex diagnoses [26]. However, research has found that truth-telling was associated with better therapeutic outcomes [24,27]. Despite this, there is a challenge in truth-telling when it is crucial to maintain hope for patients, particularly in palliative and end-of-life care issues. As such, a balance must be struck between preserving hope and telling the truth [28,29]. Another pharmacy-relevant undisclosed information is the adverse effects of medicines, with the justification that this disclosure would jeopardize adherence to drugs. Research has shown that providing information about adverse effects improves adherence to medicines [30]. In the present study, pharmacists and student pharmacists were committed to truth-telling for patients, even if physicians withheld information about adverse drug events. Truth-telling also facilitates the utilization of shared decision-making and informed consent approaches, which, to be implemented accurately, require well-informed patients [31,32]. In the present study, a discrepancy has been found between the perception of lying among pharmacy students and pharmacists. Pharmacy students report that lying is unacceptable in all circumstances, whereas pharmacists believe that lying is sometimes necessary to survive. This may be attributed to the greater exposure to professionalism, ethics, and legal courses that pharmacy students experience [17]. If pharmacists sought further education, a better understanding of the application of ethical principles could be realized [33].

It is imperative that the medicines are dispensed per ethical principles to maintain the health and safety of patients [34]. It is essential that the medicines are dispensed in accordance with ethical principles to maintain the health and safety of patients [35]. In the present study, the results revealed that pharmacy students reported better attitudes regarding the ethical dispensing of medicines compared with pharmacists surveyed. Both pharmacists and pharmacy students agreed on the importance of protecting personal health information and the problems surrounding promotional activities in pharmacies. Overall, medication errors could occur, particularly at the dispensing stage. In addition, medication-related problems could be incurred, and such issues can jeopardize achieving and maintaining therapeutic outcomes [36]. It is generally well-known that pharmaceutical marketing activities have a direct impact on physicians prescribing behavior in an ethically uncontrolled manner [37]. The protection of personal health information is mandated both lawfully and ethically, and both pharmacist students and pharmacists have positive attitudes towards the protection of patient privacy. Pharmacists agreed with the belief that results are more important than manners, whereas student pharmacists did not agree. A way to address this issue was the development and dissemination of a code of ethics, which can guide toward the ethically best practices [38]; in the present study, the student pharmacist had more awareness of the code of ethics compared to pharmacists.

Most of the items in the present study describe ethical dilemmas that are often normalized as valuable to business; however, they can also threaten patient safety or compromise other competing interests [39]. The occurrence of such behavior reflects the high number of pharmacies with regard to the population, which may negatively impact sales [40]. Another factor that may explain the occurrence of these concerns is the issues with governmental oversight of pharmaceutical institutions [39]. Additionally, the presence of unqualified non-pharmacists operating pharmacies exacerbates this issue [41]. In the present study, many ethical issues were viewed differently by pharmacists and student pharmacists, with student pharmacists exhibiting better attitudes compared to pharmacists surveyed. The recent introduction of ethical courses in the pharmacy curriculum improved the professionalism in practice for students [42]. Those practicing pharmacists may not have the same level of exposure to training, which may require pharmacists to be offered continuous education in a structured approach to ethics, focusing on ethical dilemmas.

Regarding the study limitations, the study’s cross-sectional design enables it to capture attitudes and awareness at a single point in time. Potential shifts in perception over time might not be reflected in this design. Moreover, the statistics are self-reported, which may introduce biases such as social desirability bias, where participants offer answers they believe are anticipated or socially acceptable rather than those that truly reflect their opinions. Moreover, even though a varied sample was gathered, it’s possible that the results won’t apply to pharmacists who practice in specialized or non-community contexts or outside of the participating institutions or regions. Furthermore, the observed disparities between students and practicing pharmacists may have been influenced by uncontrolled factors such as differences in educational exposure, institutional curricula, or work conditions. Nevertheless, despite these drawbacks, the study provides valuable preliminary information that might guide the establishment of ethical education and policy in pharmacy practice.

## 5. Conclusions

In conclusion, this prospective cross-sectional study emphasizes the importance of ethical awareness and attitudes among Jordanian pharmacy students and professional pharmacists. According to the results, there is a notable difference in the attitudes of professionals and students, with pharmacy students showing a stronger regard for professional ethics. The study indicates that student pharmacists typically do better in terms of ethical awareness, which is noteworthy. This suggests that exposure to academic instruction may help students develop a deeper understanding of ethical principles beyond just practical experience. These findings underscore the importance of ongoing ethics education and training for both pharmacy students and practicing pharmacists, ensuring that all professionals in the field maintain a high standard of moral behavior in patient care.

## Figures and Tables

**Table 1:** Study Sample Characteristics (n = 1737).

Factor	Category	Students (n = 1043) n (%)	Pharmacists (n = 691) n (%)
Age	<24	989	150
	≥24	54	541
Gender	Male	257 (24.6)	222 (32.1)
	Female	786 (75.4)	469 (67.9)
University	Public	51 (4.9)	570 (82.5)
	Private	992 (95.1)	121 (17.5)
Study year	≤3	349 (33.5)	
	4th year	265 (25.4)	
	5th year	429 (41.1)	
GPA	Fair	31 (3.0)	14 (2.0)
	Good	377 (36.1)	188 (27.2)
	Very good	488 (46.8)	326 (47.2)
	Excellent	147 (14.1)	163 (23.6)
Duration of training	None	352 (33.7)	25 (3.6)
	<2 months	189 (18.1)	21 (3.0)
	2–4 months	232 (22.2)	38 (5.5)
	>4 months	270 (25.9)	607 (87.8)
Have studied a course about ethics	Yes	559 (53.6)	581 (84.1)
	No	484 (46.4)	110 (15.9)
Grade of ethics course	I have not studied an ethics course	484 (46.4)	111 (16.1)
	<80%	148 (14.2)	161 (23.3)
	≥80%	411 (39.4)	419 (60.6)
Work area	Does not work	Student 768 (73.5)	9 (1.4)
	Community pharmacy	243 (23.3)	549 (79.4)
	Hospital	16 (1.5)	83 (12.0)
	Industry	12 (1.2)	38 (5.5)
	Company/drug store	1 (0.1)	8 (1.2)
Life situation Live with family	Yes	966 (92.6)	593 (85.8)
	No	77 (7.4)	98 (14.2)
The course covered all areas of ethics	Yes	747 (71.6)	509 (73.7)
	No	296 (28.4)	182 (26.3)

**Table 2:** Attitude toward ethics of pharmacy practice (n = 1737).

No	Factor	Category	Students n (%)	Pharmacists n (%)	*p*-Value
A1	Do you read independently or engage in extracurricular activities related to ethical issues in pharmacy?	Yes No	611 (58.6) 432 (41.4)	417 (60.3) 274 (39.7)	0.464
A2	In your opinion, is ethics required?	Yes No	1017 (97.5) 26 (2.5)	684 (99.0 7 (1.0)	0.027 *
A3	Honesty is the best policy	Yes No	995 (95.4) 48 (4.6)	666 (96.4) 25 (3.6)	0.318
A4	Goodness is more important than other	Yes No	971 (93.1) 72 (6.9)	657 (95.1) 34 (4.9)	0.092
A5	Lying to survive ought to be done	Yes No	199 (19.1) 844 (80.9)	101 (14.6) 590 (85.4)	0.016 *
A6	The result is more important than the process	Yes No	413 (39.6) 630 (60.4)	208 (30.1) 483 (69.9)	<0.001 *
A7	corruption will be permitted if it brings the most benefit to society	Yes No	111 (10.6) 932 (89.4)	48 (6.9) 643 (93.1)	0.009 *
A8	It’s common to ignore other people being exploited.	Yes No	488 (46.8) 555 (53.2)	276 (39.9) 415 (60.1)	0.005 *
A9	To do as most people, even if it is a wrong thing, because they do not want to be a black sheep	Yes No	152 (14.6) 891 (85.4)	59 (8.5) 632 (91.5)	<0.001 *

* p-Value < 0.05. The Cronbach’s alpha is 0.745, and the interclass correlation coefficient is 0.651.

**Table 3:** Awareness about ethics of pharmacy practice (n = 1737).

No	Factor	Category	Students n (%)	Pharmacists n (%)	*p*-Value
W1	I have a good awareness of the pharmacy code of ethics.	Strongly agree Agree Neutral Disagree Strongly Disagree	29 (2.8) 64 (6.1) 256(24.5) 272 (26.1) 422 (40.5)	10 (1.4) 29 (4.2) 89 (12.9) 140 (20.3) 423 (61.2)	<0.001 *
W2	I allow others to perform the pharmacist’s role.	Strongly agree Agree Neutral Disagree Strongly Disagree	487 (46.7) 118 (11.3) 182 (17.4) 118 (11.3) 138 (13.2)	397 (57.5) 82 (11.9) 65 (9.4) 46 (6.7) 101 (14.6)	<0.001 *
W3	The primary aim of the pharmacy is profit, and it is more important than adhering to ethical principles when practicing.	Strongly agree Agree Neutral Disagree Strongly Disagree	507 (48.6) 197 (18.9) 198 (19.0) 76 (7.3) 65 (6.2)	377 (54.6) 142 (20.5) 97 (14.0) 33 (4.8) 42 (6.1)	0.009 *
W4	I can dispense a prescription medicine without a prescription.	Strongly agree Agree Neutral Disagree Strongly Disagree	475 (45.5) 169 (16.2) 216 (20.7) 100 (9.6) 83 (8.0)	315 (45.6) 88 (12.7) 128 (18.5) 70 (10.1) 90 (13.0)	0.004 *
W5	It is important to tell the truth to the patient (including adverse drug information) even if concealed by the treating physician.	Strongly agree Agree Neutral Disagree Strongly Disagree	85 (8.1) 84 (8.1) 228 (21.9) 214 (20.5) 432 (41.4)	47 (6.8) 36 (5.2) 126 (18.2) 117 (16.9) 365 (52.8)	<0.001 *
W6	I think a pharmacist doesn’t need to be always in the pharmacy.	Strongly agree Agree Neutral Disagree Strongly Disagree	582 (55.8) 130 (12.5) 144 (13.8) 86 (8.2) 101 (9.7)	433 (62.7) 103 (14.9) 68 (9.8) 44 (6.4) 43 (6.2)	0.001 *
W7	I think it is unfavourable to share patients’ information with patients’ relatives and friends.	Strongly agree Agree Neutral Disagree Strongly Disagree	72 (6.9) 71 (6.8) 211 (20.2) 161 (15.4) 528 (50.6)	54 (7.8) 44 (6.4) 88 (12.7) 99 (14.3) 406 (58.8)	0.001 *
W8	I would dispense a prescription that has an error without contacting the doctor.	Strongly agree Agree Neutral Disagree Strongly Disagree	697 (66.8) 123 (11.8) 107 (10.3) 55 (5.3) 61 (5.8)	531 (76.8) 66 (9.6) 44 (6.4) 26 (3.8) 24 (3.5)	<0.001 *
W9	It is okay for me to recommend alternative drugs because they are more expensive.	Strongly agree Agree Neutral Disagree Strongly Disagree	423 (40.6) 191 (18.3) 236 (22.6) 109 (10.5) 84 (8.1)	338 (48.9) 138 (20.0) 138 (20.0) 45 (6.5) 32 (4.6)	<0.001 *
W10	In today’s competitive work environment, it is possible to have promotional activities in the pharmacy.	Strongly agree Agree Neutral Disagree Strongly Disagree	60 (5.8) 101 (9.7) 328 (31.4) 273 (26.2) 281 (26.9)	35 (5.1) 43 (6.2) 175 (25.3) 164 (23.7) 274 (39.7)	<0.001 *
W11	I can recommend to patients several non-medical health products other than medicine.	Strongly agree Agree Neutral Disagree Strongly Disagree	130 (12.5) 95 (9.1) 283 (27.1) 283 (27.1) 252 (24.2)	117 (16.9) 74 (10.7) 155 (22.4) 174 (25.2) 171 (24.7)	0.024 *
W12	To maintain the well-being of patients, we should not disparage the skills of other healthcare professionals.	Strongly agree Agree Neutral Disagree Strongly Disagree	491 (47.1) 169 (16.2) 211 (20.2) 91 (8.7) 81 (7.8)	374 (54.1) 129 (18.7) 106 (15.3) 39 (5.6) 43 (6.2)	0.001 *
W13	I may dispense an OTC medication; the customer requested an OTC medication despite you having doubts that he is abusing it.	Strongly agree Agree Neutral Disagree Strongly Disagree	618 (59.3) 137 (13.1) 170 (16.3) 54 (5.2) 64 (6.1)	490 (70.9) 94 (13.6) 59 (8.5) 26 (3.8) 22 (3.2)	<0.001 *
W14	I can supply Natural products with undocumented safety and efficacy if requested by the patient.	Strongly agree Agree Neutral Disagree Strongly Disagree	449 (43.0) 84 (19.5) 221 (21.2) 84 (8.1) 86 (8.2)	336 (48.6) 141 (20.4) 112 (16.2) 51 (7.4) 51 (7.4)	0.060

* p-Value < 0.05. The chi-square test was used in this Table.

## Data Availability

The data underlying this article will be shared on reasonable request to the corresponding author.
